# Abstract concepts and simulated competition

**DOI:** 10.1007/s00426-023-01843-7

**Published:** 2023-06-03

**Authors:** Daniele Nico, Anna M. Borghi, Luca Tummolini, Elena Daprati

**Affiliations:** 1 Department of Psychology, Sapienza University of Rome, Via dei Marsi 78, 00185 Rome, Italy; 2 Department of Dynamic and Clinical Psychology, and Health Studies, Sapienza University of Rome, Rome, Italy; 3 Institute of Cognitive Sciences and Technologies, Italian National Research Council, Rome, Italy; 4 Institute for Future Studies, Stockholm, Sweden; 5 Dipartimento di Medicina dei Sistemi and CBMS, Università di Roma Tor Vergata, Rome, Italy

## Abstract

To better understand the social determinants of conceptual knowledge we devised a task in which participants were asked to judge the match between a definition (expressed in abstract or concrete terms) and a target-word (also either abstract or concrete). The task was presented in the form of a competition that could/could not include an opponent, and in which different percentages of response rounds were assigned to the participant at the experimenter’s discretion. Thus, depending on the condition, participants were either exposed to a competitive context mimicking a privileged/unprivileged interaction with the experimenter or to a socially neutral setting. Results showed that manipulation of the social context selectively affected judgments on abstract stimuli: responses were significantly slower whenever a definition and/or a target word were presented in abstract form and when participants were in the favorable condition of responding in most of the trials. Moreover, only when processing abstract material, responses were slower when an opponent was expected to be present. Data are discussed in the frame of the different cognitive engagements involved when treating abstract and concrete concepts as well as in relation to the possible motivational factors prompted by the experimental set-up. The role of social context as a crucial element for abstract knowledge processing is also considered.

## Introduction

In his famous Dialogues, Plato imagines his master Socrates stating that: “a good decision is based on knowledge and not on numbers” (in Jovett, 1931: “The dialogues of Plato”, Laches, p. 91). Apart from any philosophical consideration, what transpires is the intention to stress the need for relying on experience and reasoning to make the proper choice. In cognitive terms this means that before selecting an optimal course of action, one must refer to representational abilities and abstraction in order, for instance, to anticipate the possible outcomes of behavior. Modern theories of cognition depict the brain as a predictive system: in these views, the products of experience guide behavior through abstract reasoning, allowing to achieve whichever goals are defined by internal states and environmental opportunities (see for instance Gilead et al., 2020). Following the principle of prediction error minimization, the ability to abstract from contingent reality (i.e., to refer to inner representations and work on them) is then a prerequisite to any decision. Indeed, abstraction allows to form representations that are detached from “concrete” sensorimotor experiences and lack perceptually bounded references (Borghi et al., 2017).

The acquisition of abstraction abilities clearly marks the development of the human brain and is strictly connected with social behavior, as testified by the most characteristic of children’s behaviors: play. Play describes all activities that share the common feature of being performed for fun, namely all actions that are not immediately related to some practical purpose or to a clearly distinguishable goal. “Pretend play” in particular, strongly relies on representational abilities. In pretend play, the child behaves in “as-if” mode, using objects as substitutes and within a context that is clearly not real. Remarkably, the degree of abstraction involved in this play changes with age: in the first form of pretend play, which emerges around 18 months, objects are used as if they were something else. In contrast, in preschool years, children achieve complete independence from reality and pretend play can be performed without physical objects and entirely with imagination (Lillard, 2017; Weisberg, 2015). Albeit a precise definition of the causal link between pretend play and several types of abstract reasoning is still a matter of debate there is a clear functional association between the symbolic activity involved in this kind of play and the mental capacity of working with abstract knowledge – such as in counterfactual thinking, theory of mind and language (Lillard & Kavanaugh, 2014; Weisberg, 2015). In this sense, playing represents a crucial step in the acquisition of the ability to symbolize but is also deeply intertwined with the development of social interactions.

Cross-cultural studies showed that pretend play does occur in a social context (such as during parent–child interaction) and involves specific signals aimed at stressing the artificial, non-literary meaning of what is performed (Ma & Lillard, 2017; Nishida & Lillard, 2007). As explicitly stated by Lillard: “pretend play is about achieving joint-attention and communicating about abstraction, about pretend behaviors that symbolize their real counterparts” (Lillard, 2017, p.830). The more pretend play involves another player the more the child is exposed to behavioral cues that emphasize the symbolic nature of the actions involved in pretending. Consequently, the social experience of playing with imagination not only promotes the ability to symbolize but also helps developing the type of social sensitivity that is required to fully understand novel communicative gestures (Ma & Lillard, 2017) and to participate in collective activities (Rakoczy, 2007). In this sense, social interaction favors the development of abstract knowledge and reasoning, because it enhances the cognitive resources required by play, especially when it involves joint action with peers (Heesen et al., 2017). Indeed, engaging with other people for a common purpose asks for complex cognitive competences such as joint attention, prediction, shared emotions and of course metarepresentations (Vesper et al., 2017), which the act of playing may further contribute to construct. In such a “social” perspective, in which play assumes a characterizing role, language is both an instrument to communicate and the basis for building the representations that conceptually define the growing complexity of reality. Interestingly, in the Diagnostic and Statistical Manual of mental Disorders (DSM) absence of shared symbolic play is now considered among the diagnostic criteria for autistic spectrum disorders (American Psychiatric Association, 2013), highlighting the intimate link between play and the development of language, social skills, and abstraction. Indeed, compared to their peers, children with autism progressively accumulate delays in the social and language domain (Lord et al., 2020) and are often impaired in making inferences about false belief and counterfactual thinking (Rasga et al., 2017).

Understanding the cognitive processes involved in creating an abstract representation of the external world is of relevance not only to the developmental domain but also to the more general comprehension of how concepts are constructed and manipulated. Recent theories of “embodied” and “grounded” cognition consider conceptual knowledge as rooted in actions and experiences (see for instance Barsalou, 1999, 2008), suggesting an interesting, dynamic interplay between concepts and the elements they represent. In this respect, abstract concepts, not being primarily grounded in the (physical) direct experience of a tangible item, represent an anomaly that can be accounted for by referring to the mediation offered by social interaction, language, and shared communication (Borghi et al., 2017). Abstract concepts (e.g., truth), although not opposed to concrete ones (e.g., shirt) in a dichotomous way, differ from them in various respects. They are less likely to elicit images; they are typically acquired later and are learned linguistically rather than perceptually. Also, the words expressing abstract concepts are less iconic, i.e., have a lower form-shape resemblance. In fact, abstract concepts appear to be more dependent on linguistic, emotional, and social aspects than concrete ones (e.g., Borghi et al., 2019; Connell et al., 2018; Cuccio & Gallese, 2018; Dove, 2020, 2022; Dove et al., 2020; Harpaintner et al., 2018; Henningsen-Schomers & Pulvermueller, 2021; Kurmakaeva et al., 2021, Vigliocco et al., 2014; Villani et al., 2021, 2022; Zdrazilova et al., 2018). Furthermore, their meaning is more variable across individuals, contexts, and cultures (Borghi, 2022), making them less determined, and likely to generate higher uncertainty (Mazzuca et al., 2022). Consequently, the feeling that others could be more relevant to understand their meaning would emerge (social metacognition, Borghi et al., 2018), as testified by rating and interactive tasks (e.g., Fini et al., 2021; Villani et al., 2022). The uncertainty in the word meanings may be solved by reverting to others (Prinz, 2014; Shea, 2018), who can help  by providing explanations and discussing their meanings,  contributing with their point of view (Fini et al., 2021). For these reasons, abstract concepts have been defined as “concepts for which we need others more” (Borghi, 2022). In this respect, and relevant to the present work, recent evidence suggests that the first comprehension of abstract concepts in infants and children may be related to the emergence of social skills, such as the ability to follow the gaze of others and to engage in joint action (Bergelson & Swingley, 2013). In this framework, the manifold connections that play has with social skills, language and abstract reasoning indicate that play could represent a powerful tool to investigate the cognitive processes involved in conceptual knowledge. In fact, being deeply embedded in that functional ‘social’ loop that links human relationships and language through symbolic ability, this peculiar form of interpersonal interaction is likely to maintain a constant role in supporting representational abilities, even in adult life.

Within the domain of play, a component that clearly brings together abstract reasoning and social skills is competition. Competition is a multifaceted condition, in which processes and responses pertaining to interpersonal relations are framed in the context of perceived rivalry, as can be encountered in every domain of life, from leisure to work. Interestingly, competition arises not only from social comparison, namely as behavior aimed at obtaining a payoff that is ‘good’ in relation to other people’s judgment or achievement but also as a more intrinsic, physiologically aroused desire to surpass the other, even if this means disregarding the effective payoff (i.e., as a mere ‘desire to win’, Malhotra, 2010). In addition to motivational aspects, competition relies on a complex pattern of abstract cognitive operations, which include decision-making, strategy planning and prospective thinking. Besides, competition undeniably requires the ability to mentalize, i.e., to understand the opponent in terms of intentional mental states, such as feelings and goals (Tsoi et al., 2016). In this perspective, it is interesting to note that winning a competition without a payoff for ourselves but causing a loss to the rival activates a set of brain structures that include a core part of the human rewards system such as the striatum, and areas crucial for social cognition and mentalizing (e.g., temporoparietal junction and the precuneus, Votinov et al., 2015). The basically social nature of competing is further emphasized by the fact that it is exactly the presence or absence of an opponent that affects brain activity. In an animal study, Hosokawa and Watanabe (2012) trained monkeys to play a computer game and recorded the activity of neurons in their lateral prefrontal cortex. Albeit their research focused on the motivational aspects of competition, results clearly showed an effect of the ‘social situation’. Playing against a conspecific (as opposed to an identical condition in which the animal played against the computer) significantly affected not only behavioral responses (namely, faster and more accurate shots) but also the corresponding pattern of neuronal activations (Hosokawa & Watanabe, 2012). More recently, Demolliens et al. (2017) recorded the activity of single neurons in monkeys engaged in a visuomotor task, either in the presence of a conspecific or alone. The animals’ responses changed when a conspecific was present, and most of their prefrontal neurons revealed a specific sensitivity to the performance context. Interestingly, two populations of neurons were detected: ‘social neurons’ and ‘asocial neurons’, the former active when the animal responded in the presence of another monkey, the latter in a context of social isolation. Of relevance here, the same task recruited either social or asocial neurons depending on the presence or absence of a conspecific (Demolliens et al., 2017). An experimental approach like that of Hosokawa was used in humans in an fMRI study (Kätsyri et al., 2013). The authors used a first-person tank-shooter videogame to contrast brain activations in two conditions: one in which the player faced tanks driven by a human opponent and one in which they were told that the enemy tanks were led by the computer. It should be noted that to have comparable levels of difficulty participants in fact always competed against the same human player, the only difference being the verbal information they received about the nature of their opponent before starting to play. As stressed by the authors, subjective awareness about the social context sufficed to alter brain responsiveness. Indeed, winning against a supposed human opponent resulted in higher activations in ventral caudate, ventromedial and dorsomedial prefrontal cortices (vmPFC, dmPFC). This differential pattern of response could reflect higher values of experienced reward linked to the emotional and cognitive aspects of interacting with a human opponent compared to a machine (Kätsyri et al., 2013), further strengthening the relevance of the social component in game playing.

To sum up, data from the developmental literature underline the role of play in mastering the symbolic abilities required for the acquisition of language, shared knowledge, and social skills. Social cognition maintains a pivotal role also in adult play, and more generally in verbal communication, providing a valuable scaffolding to read and predict other people’s intentions and properly contextualize the meaning of their words. This latter aspect is particularly relevant for certain concepts, such as abstract ones. In this respect, the Words As social Tools (WAT) theory (Borghi et al., 2019) argues that social experience is especially crucial for abstract concepts because of their open and indeterminate character. Notably, other people can support and scaffold the acquisition of abstract concepts (e.g., Bergelson & Swingley, 2013), providing information on their meaning to facilitate their comprehension, or negotiating the assigned meaning if diverging on its interpretation (Borghi, 2022). Besides, when abstract concepts are concerned, social skills contribute to language communication, enabling individuals to assess the person they are dealing with, inferring and/or modulating the subtle nuances of meaning that may better suit his/her status. Indeed, the same concept can acquire different meanings based on the age and experience of the individual using it (e.g., Buccino et al., 2019). Accordingly, it is reasonable to assume that situations that strongly call for social skills and representational abilities may differently interact with the processing of abstract vs. concrete concepts. On these bases, the present study was designed to test whether and how social interaction impacts abstract concept processing. In fact, while various studies have addressed the role of linguistic knowledge in abstract concepts, the influence of social experience has yet to be extensively investigated (for exceptions, see Fini et al., 2021, 2023; Pexman et al., 2023).

We used a task of simulated competition to test how the verbal processing of concepts responds to changes in the degree of social and emotional commitment implied by the task. Healthy volunteers were asked to decide—as rapidly and accurately as possible—whether a definition and a word matched or not. They were told that points were assigned based on the number of correct responses provided. The effects of abstractness of the presented material and the degree of social and emotional involvement were assessed by introducing the following manipulations. Abstractness was varied by including stimuli in which the definition, the target concept, or both items were either abstract or concrete. Social and emotional involvement were explored by devising conditions that differed in terms of competitive environment and fairness of distribution of response rounds. The competitive component was manipulated by assigning half of the participants to a 2-players condition, and the other half to a condition in which they played alone. Fairness of social support was varied by informing participants in the 2-players condition that the assignment of rounds of response would be entirely at the experimenter’s discretion. This information—together with the notion that points were credited based on correct responses—was expected to produce conditions in which the experimenter appeared to either support or obstruct the current participant.

Based on previous literature (e.g., Demolliens et al., 2017; Hosokawa & Watanabe, 2012; Kätsyri et al., 2013; Tsoi et al., 2016) we anticipated that game playing—especially in the most competitive condition—would recruit processes involved in both social behavior and abstract reasoning. At this stage, we did not advance predictions on whether the effects of the manipulation would emerge as selective facilitation or interference with the processing of abstract material. The literature provides many an example that tasks loading on the same functional modules can both facilitate (e.g., McNair & Harris, 2012 for an example of motor priming effects; Jausovec & Habe, 2005 for an example on music facilitation of spatial processing) and hinder performance (e.g., Hartikainen et al., 2000; Nico & Daprati, 2015 for examples in the visuospatial domain; Chersi et al., 2010, García & Ibáñez, 2016 for examples of interference and facilitation during language processing). Nonetheless, we expected to find a difference between concrete vs. abstract material in response to the manipulation. Namely, if social interaction is relevant to the processing of abstract material, differences should emerge in the participants’ performances according to the conditions they are assigned to. In addition, we anticipated that the combinations richer in abstract elements (i.e., abstract concepts defined in abstract terms) would be most sensitive to the manipulations introduced.

## Methods

### Participants

The study was conducted in accordance with local ethical committee guidelines. All participants gave informed consent before starting the experimental session. Due to the Coronavirus pandemic, the entire experiment was carried out online. Participants were invited to join via advertisements posted on students’ groups on the major social networks (e.g., Instagram, WhatsApp, etc.). Inclusion criteria were a) Italian as a first language; b) negative history of neurological and/or psychiatric diseases; c) not having taken medicaments interfering with alertness (e.g., antihistamines) in the past 24 h. A simulation using G-power (Faul et al. 2007) assuming alpha = 0.05 and power = 0.8, suggested a total sample size of 60 participants (72 participants for power = 0.9; i.e., 10–12 participants per condition). Compared to standard laboratory settings, the percentage of dropouts is expected to be larger in online studies because participants are less affected by situational demands (e.g., feeling of obligation) and more likely to abandon the study before completion (Crump et al., 2013; Dandurand et al., 2008). Besides, the possibility of encountering technical difficulties or commit procedural errors is inevitably increased compared to a more supervised setting, implying that more participants could be discarded in the phase of data analyses due to errors or data loss. To account for these limitations, we initially contacted between 16 and 18 participants for each condition. Of the 105 volunteers that agreed to participate, sixteen were discarded because they did not meet the inclusion criteria or because they did not complete the experimental session. Seven participants were further discarded in the phase of data analyses due to technical or procedural errors (i.e., partial data loss; use of incorrect keys). The current dropout rate (approx. 20%) is compatible with what is reported in the literature (Crump et al., 2013; Dandurand et al., 2008). The eighty-two participants who correctly completed the task (age *M* = 24.1, SD = 2.8, education *M* = 15.7, SD = 1.9, 59% females, 5% left-handers) all complied with inclusion criteria and were naive as to the purpose of the study. The number of participants assigned to each condition is reported in Table 1.

**Table1:** Summary of the experimental conditions

Task presented as involving…	Response frequency	Response trials/wait trials (%)	*N*
Opponent +			
2-players	High	67/33	15
2-players	Equal	50/50	15
2-players	Low	33/67	16
Opponent−			
1-player	High	67/33	11
1-player	Equal	50/50	12
1-player	Low	33/67	13

Participants were assigned to one of six experimental conditions based on recruitment order. Participants in the Opponent + groups were informed that they would play against another participant (2-players), whereas participants in the Opponent- groups were told they would perform individually (1-player). In truth, a second player was never present. All participants responded to the same number of trials (‘response’ trials). Conversely, the number of ‘wait’ trails varied according to condition. Participants in the High condition were kept on hold less frequently than participants in the Equal and Low conditions (who were administered twice the amount of ‘wait’ trials experienced by those in the High condition). The number of participants included in each condition is reported in the rightmost column (*N*)

### Stimuli

Stimuli were word/definition pairs. Both words and definitions were presented on the computer screen as white text (font: sans-serif 30) on a black background. The definition was displayed in the upper half of the screen, the word in the lower half (0,150; 0,− 150 respectively; coordinates referring to the center of the image; 0,0 being the center of the screen). More details on word/definition pairs and their selection are given in Appendix 1.

#### Words

Forty words were selected from a large database of Italian words (Della Rosa et al., 2010). Twenty were concrete words (Cw: flag, bicycle, sock, hat, helmet, cave, helicopter, oak, sand, statue, cement, shell, tie, fountain, ice, jacket, armchair, boot, drill, tractor), and twenty were abstract words (Aw: philosophy, justice, irony, liberty, merit, principle, reason, style, tendency, concept, conscience, criticism, culture, fate, judgment, instinct, logic, motive, originality). Words with strong emotional content were purposefully excluded. Separate t tests showed that concrete and abstract words were comparable in terms of Length and Familiarity but significantly differed along the dimension of interest, Concreteness and Abstractness (see Table2) (for a description of the dimensions and relative values, see Della Rosa et al., 2010).

**Table 2:** (A) Parameters relative to the concrete and abstract words included in the study

(A) All words	Concrete words	Abstract words	p
Length (letters)	7.4 (1.5)	7.5 (1.4)	0.48
Familiarity	552 (63)	533 (39)	0.26
Concreteness	682 (16)	224 (45)	< 0.0001
Abstractness	109 (9)	553 (41)	< 0.0001

(B) Words in set 1 and 2	Set1	Set2	*p*
Length (letters)	7.3 (1.5)	7.6 (1.3)	0.41
Familiarity	541 (63)	544 (42)	0.87
Concreteness	452 (234)	453 (241)	0.92
Abstractness	330 (224)	332 (235)	0.84

(C) Definitions in set1 and 2	Set1	Set2	*p*
Length (syllables)	23.8 (6.5)	23.2 (6.5)	0.72
Word production	0.69 (0.24)	0.75 (0.21)	0.21
Word/definition association	5.58 (0.58)	5.48 (0.66)	0.51
Concreteness/Abstractness rating	3.90 (0.92)	3.99 (0.92)	0.56

Mean values (SD) relative to Length, Familiarity, Concreteness and Abstractness for the 20 concrete and 20 abstract words included in the study (based on Della Rosa et al., 2010). (B) Comparisons between words included in set 1 and 2. Mean values (SD) relative to Length, Familiarity, Concreteness and Abstractness ratings for the 10 concrete and 10 abstract words included in set1 and 2. (C) Comparisons between definitions included in set 1 and 2*.* Mean values (SD) relative to Length, Word production, Word/definition association, Concreteness/Abstractness ratings for the definitions included in set1 and 2. P-values relative to separate *t* tests comparing the two types of words and the two sets are also reported

#### Definitions

For each word, two different definitions were created, for a total of 80 definitions. One definition, i.e., concrete definition (Cd), described the concept mainly by means of perceptual attributes or real-world examples. Conversely, the second definition, i.e., abstract definition (Ad) described the target concept using a theoretical framework or taxonomic relations. For example, the two definitions for ‘freedom’ were as follows: Cd, “Those in prison or in slavery are deprived of it”; Ad, “The condition of a person not subject to constraints”. A pre-test was run to ensure that the selected definitions correctly captured the intended content and that Cd and Ad were perceived as different by the readers (see details in Appendix 1).

#### Word/definitions pairs

Of these 80 word/definition pairs (Matching Pairs, MP), half were combinations in which both the word and the terms of the definition were either Abstract (Aw/Ad) or Concrete (Cw/Cd), the other half were combinations in which an Abstract concept was described by a Concrete definition (Aw/Cd), and vice versa (Cw/Ad). This was done to manipulate the amount of abstract material participants were asked to deal with. Using the same words and definitions, an equal number of pairs was constructed in which the definition did not match the concept described by the word (Not-Matching Pairs, NMP). Again, the accompanying word always belonged in the same category (e.g., if the correct target was an abstract word, the not-matching item was also an abstract word) and was randomly extracted from the same pool as the matching ones. Matching pairs required a “yes” response; Not-Matching pairs required a “no” response (see procedure). Not-matching pairs were introduced to explore RTs relative to both affirmative and negative responses. This was done because if present, differences in latency between the two types of responses may inform the operations leading to the decision.

To keep task duration to a minimum, the 160 pairs so obtained were split into two sets of stimuli, each containing 40 matching pairs (i.e., 10 concrete words, each paired with one concrete and one abstract definition; 10 abstract words, each paired with one concrete and one abstract definition), and 40 not-matching pairs (equally distributed). Separate t-tests confirmed that words included in Set 1 and 2 did not differ with respect to Length, Familiarity, Concreteness, and Abstractness. The same was true for definitions, which did not differ in terms of Length Word production, Word/definition association, and Concreteness/Abstractness ratings (see Table 2). Administration of set 1 and set 2 was balanced across participants. Within each set, in separate blocks words were presented in combination with the abstract and concrete definition. Order of blocks was counterbalanced between participants to control for possible carry-over effects.

### Procedure

The experiment was entirely carried out online. Assistance throughout the session was ensured by one experimenter, who kept in touch with the participant by means of the chat on his/her preferred social media (e.g., WhatsApp, Instagram, etc.).

#### Software

The software PsyToolkit was used (Stoet, 2010, 2017). Results obtained with this platform are reported to consistently replicate those collected in the lab using commercial software for cognitive neuroscience experiments. This is particularly true for studies using linguistic stimuli and collecting response choices and response times (Kim et al., 2019)–as is the case here. To respect privacy issues, users’ IP address and location information were never stored. Mobile phone and tablet users were excluded because a real keyboard was required for the responses.

#### General organization of the experimental session

All participants were informed that they would perform a task requiring a decision on the congruence between a definition and a target-word (cf. Borghi & Zarcone, 2016). On the day of the experiment, they received an email containing the link to access the online platform used for the study. At the agreed time, participants were contacted by one experimenter and asked to access the website to read and fill in the informed consent form. After accepting to join in, they were redirected to a page requesting to enter a one-time password, which they could obtain from the experimenter via the chosen chat. This was done to make sure that participants received answers to any questions they may have before entering the experimental session and to avoid that they accessed it before the agreed time. Upon entering the password, the checklist relative to the inclusion criteria appeared on the screen. If participants did not meet these criteria, they were thanked for their help and the online session was automatically terminated. Conversely, if they met the criteria for inclusion, they were presented with a questionnaire aimed at acquiring basic demographic information (age, sex, education, manual preference). When the questionnaire was finished, detailed instructions for the task were presented on the screen. These included the information that participants could gain points based on their performance, and that at the end of the study the three participants that had obtained the highest scores would receive a gift voucher for an online store. In fact, the competition was a ruse, only meant at making the task more engaging. Once participants had read the instruction webpages, they were asked once more to contact the experimenter, who was available for questions or–in case all was clear, would give them the go-signal. The experimental session began with 8 training trials, which were followed by two blocks, each containing 40 experimental trials. A pause was offered between the two blocks, with a message asking participants to inform the experimenter about when they wished to resume the task. At the end of the second block, a short debriefing session was run. A series of questions appeared on the screen that inquired about how participants had perceived the task. Finally, a text box for free comments appeared. This completed the experimental session and terminated data collection, exiting the PsyToolkit platform.

All participants were told they were welcome to reconnect with the experimenter at the end of the session should they have any questions or simply to say good-bye. Participants wishing to remain anonymous were presented with a unique code that they could use as a reference in case they needed to contact the researchers. Average duration of the session was approx. 30–40 min.

#### Trial structure

In all conditions, there were two sets of trials: “response” trials and “wait” trials. In “response” trials, participants were asked to decide whether a definition was correct for the target-word. In “wait” trials they remained idle.

**Fig. 1:**
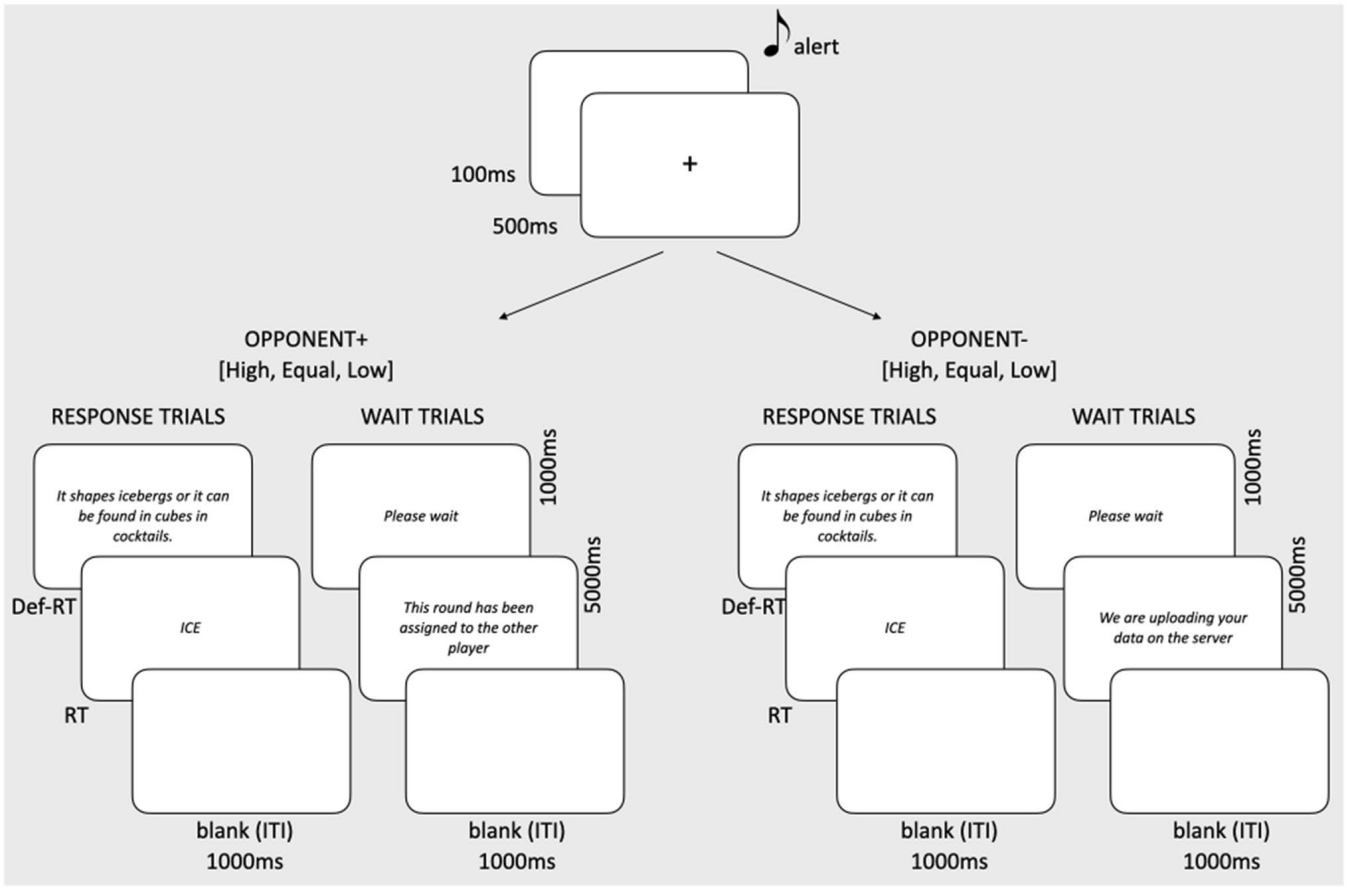
Trial structure and relative timing. There were two sets of trials: “response” trials and “wait” trials. In “response” trials, participants were asked to decide whether a definition was correct for the target word. In “wait” trials participants were kept on hold, for the reason that the other player was responding (Opponent + conditions) or that their data were being uploaded on the server (Opponent− conditions). The task for all participants was to carefully read the definition and press the space bar only when they had finished reading it (Def-RT). Upon key press, a word appeared, and participants had to decide whether it matched/not matched the definition. They pressed the “*b*” and “*n*” keys on their keyboard for “yes” and “no” responses respectively (RT)

A description of the different trials’ structure is given in Fig. 1. Briefly, all trials began with a black screen and a brief alert sound (100 ms). Next, a white fixation cross appeared in the center of the screen and remained visible for 500 ms. In “response” trials, this was followed by the appearance of the definition. Participants were asked to carefully read the definition and press the space bar only when they had finished reading. Upon key press, a word appeared, and participants had to decide whether it matched/not matched the definition. They pressed the “*b*” and “*n*” keys on their keyboard for “match” and “not match” responses respectively. Following the key press, both the definition and word disappeared, a black screen was presented for 1 s (ITI), and the next trial started.

Participants were asked to respond as rapidly and accurately as possible, as both measures would contribute to their final score (and increase their opportunity to gain points). On the instruction page, it was also specified that the index and middle finger of the right hand should be used to provide respectively the yes and no response, while the index finger of the left hand should be used to press the space bar. On a QWERTY keyboard (the most widely used keyboard in Italy), the “*b*” and “*n*” keys are located centrally, making it comfortable for the participants to provide both responses without moving the right hand excessively.

#### Experimental conditions

A between-subjects design was applied to minimize confounds due to repetitive exposure to the test material. Participants were randomly assigned to one of six experimental conditions. A summary of the conditions is given in Table 2, where the final number of participants for each group is also reported.

To explore the effects of being involved in direct competition, in three conditions (Opponent +) participants were falsely led to believe that a second player was present and that they were actively competing with him/her. To keep up this pretense, the experimenter presented the task as requiring two players and made as if the rendezvous day and time had to be arranged to suit both participants. On the day of the experiment, before giving the go-signal, the experimenter pretended to check whether the other player was also online and repeated this pantomime when the task had to be resumed after the pause. To test the effect of social support, in all Opponent + conditions, participants were told that turns of response were decided by the experimenter who could choose, trial-by-trial, to whom to assign the next round. It was emphasized that this choice was entirely at the experimenter’s discretion. Three combinations of “response” and “wait” trials were tested in a between-subjects design. In one condition (High), the proportion of “response” trials was high and that of “wait” trials was low: namely, the response round was assigned to the current participant in two-thirds of trials (67%), and to the fictional, second participant in one-third of trials (33%). Accordingly, participants assigned to this condition were unfrequently kept on hold (they seemed to receive most of the ‘response’ trials), as if the experimenter supported them by giving them more opportunities to gain points. Conversely, in the complementary condition (Low), participants responded in 33% of trials and were kept on hold in 67% of trials. Accordingly, these participants were less frequently called to respond being kept on hold for longer periods, as if the experimenter neglected and/or obstructed them favoring their competitor. Finally, in a third condition (Equal) the response round was assigned to the current participant in 50% of trials and to the fictional second player in the remaining 50% (i.e., the participant was kept on hold in half of the trials), simulating a fair behavior on the part of the experimenter.

In three other conditions (Opponent-), the task was described as involving one participant at a time. This was done to control for possible non-specific effects derived from being involved in a task in which speed and accuracy were emphasized and for the fact of being kept idle for different amounts of time in the High, Equal and Low conditions (in which the number of ‘wait’ trials varied from 33 to 67%). The role of the experimenter was presented to these participants as that of a guide or an assistant in case any issue would arise during the task. The same combinations of ‘response’ and ‘wait’ trials described for the Opponent + groups were applied (High, Equal, Low) but—as there was no second player—when kept on hold, participants read a message informing that data were being uploaded to the server.

All participants were administered the same number of ‘response’ trials, the difference between the High, Equal and Low conditions referring only to the number of ‘wait’ trials assigned to the current participant (i.e., the time he/she spent being kept on hold). Despite what was told to participants, data uploading never delayed the task and a second player was never present. In both Opponent + and Opponent- conditions the percentage of trials in which they were called to respond was identically manipulated. The only difference between the Opponent + and Opponent- conditions related to the message participants received.

### Data collection and analyses

For each participant, the program created two files, one relative to the questionnaires (demographic data and debriefing section), and the other relative to the experimental task. The two files were separately analyzed so that the person working on the data was blind as to the identity of the participant and the group he/she belonged to. Throughout the experimental session, the experimenter could not see the participant’s responses.

#### Collected measures

Responses were considered correct if participants pressed “b” (“match”) when the definition matched the target word, and “n” (“not match”) when the definition and word did not match. The proportion of correct responses out of the total number of presented items was computed for each participant and each stimulus category. Proportions were submitted to arcsine transformation prior to being submitted to parametric analyses. In addition, for each participant, two latency measures were collected, time spent on the definition (Def-RT) and time required to respond (RT). Def-RT was the time elapsed between the appearance of the definition on the screen and the first key press (space bar). RT was the time elapsed between the appearance of the word and the second button press (“*b*” for match and “*n*” for not match). Def-RT was assumed to reflect the time spent reading and processing information relative to the definition, RT the time devoted to reaching a decision on the possible match between word and definition. For both measures, responses faster/slower than the overall participant’s mean ± 2 standard deviations (2%) were removed before entering data in the analyses. Responses to a short set of debriefing session were also collected and explored via descriptive statistics.

### Statistics

Proportion of correct responses and average Def-RT scores were submitted to separate ANOVAs with Word (Aw, Cw) and Definition (Ad, Cd) as within-subject factors, and Opponent (Opponent + , Opponent−) and Proportion (High, Equal, Low) as between-subject factors. RTs were submitted to an ANOVA with Word (Aw, Cw), Definition (Ad, Cd), and Pair (Matching Pair MP, Not Matching Pair NMP) as within-subject factors, and Opponent (Opponent + , Opponent−) and Proportion (High, Equal, Low) as between-subject factors. Newman-Keuls test was used for post-hoc comparisons whenever appropriate. For all statistics, the alpha level for acceptance was set at 0.05. Bonferroni correction was applied in the case of multiple comparisons.

## Results

In each trial, participants spent approx. 70% of the time on the definition and the remaining 30% deciding whether it matched the appearing word. This distribution was similar across the different groups (Opponent-: High 68–32%, Equal 66–34%, Low 67–33%; Opponent + : High 63–37%, Equal 68–32%, Low 67–33%), testifying that all participants correctly followed the instruction to carefully read the definition before calling for the word.

### Accuracy

Overall, accuracy was very high, with correct responses being approx. 95% in all groups (Opponent-: High 94%, Equal 97%, Low 94%; Opponent + : High 95%, Equal 95%, Low 94%). The ANOVA with Word (Aw, Cw) and Definition (Ad, Cd) as within-subject factors, and Opponent (Opponent + , Opponent−) and Proportion (High, Equal, Low) as between-subject factors showed only the main effects of Word, F(1,76) = 45.27, *p* = 0.000001 and Definition, F(1,76) = 7.52, *p* = 0.008. As expected, accuracy was higher when participants responded to concrete words (Cw, *M* = 0.97, SD = 0.04) than to abstract words (Aw, M = 0.93, SD = 0.05), and to concrete definitions (Cd, M = 0.96, SD = 0.04) than to abstract definitions (Ad, *M* = 0.94, SD = 0.05). The main effects of Opponent and Proportion were not significant and there were no significant interactions (all *p* > 0.05).

### Time spent on the definition (Def-RT)

**Fig. 2:**
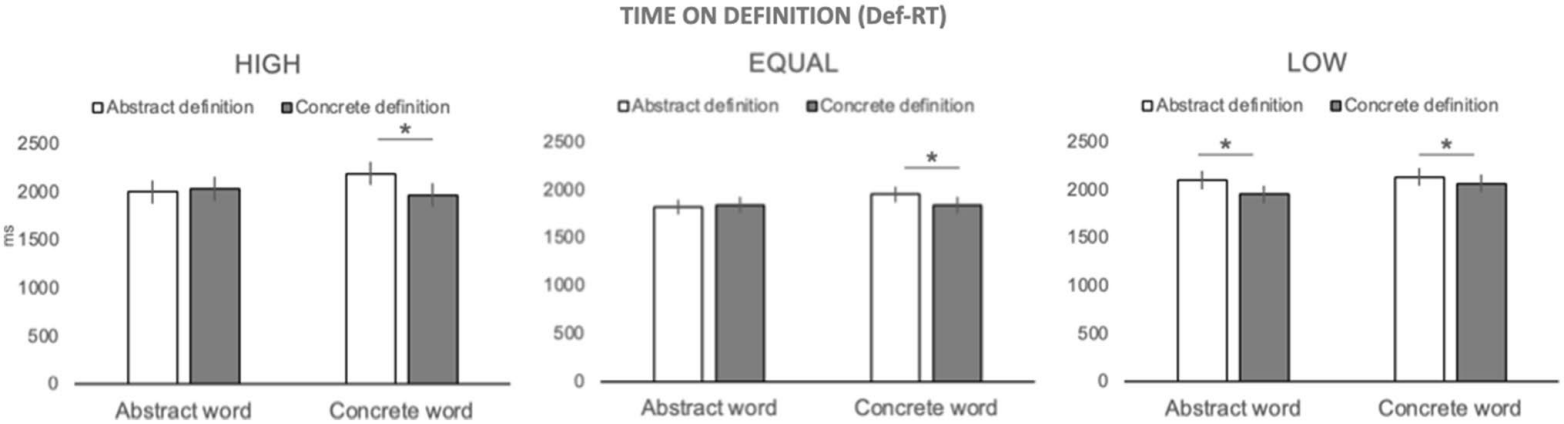
Time spent on the definition (Def-RT). Def-RT was the time elapsed between the appearance of the definition and the first key press, i.e., the key press aimed at making the word appear on the screen. In all conditions, longer Def-RTs were recorded for abstract definitions of concrete words than for the corresponding concrete definitions. In addition, for participants in the Low condition, a significant difference between abstract and concrete definitions emerged also for abstract words. Labels as follows: High, 67% of trials were “response” trials, 33% were “wait” trials; Equal, 50% of the trials were “response” trials, 50% were “wait” trials; Low, 33% of trials were “response” trials, 67% were “wait” trials. Whiskers refer to standard errors; asterisks indicate significant comparisons (*p* < 0.05)

Average time spent reading the definition was approx. 2 s (*M* = 1993 ms, SD = 486 ms). The ANOVA with Word (Aw, Cw) and Definition (Ad, Cd) as within-subject factors, and Opponent (Opponent + , Opponent−) and Proportion (High, Equal, Low) as between-subject factors showed the main effects of Word, F(1,76) = 8.47, *p* = 0.005 and Definition, F(1,76) = 6.38, *p* = 0.014. The related interaction Word x Definition was also significant, F(1,76) = 14.41, *p* = 0.0003 as was the higher-order interaction between Proportion, Word and Definition, F(2,76) = 14.12, *p* = 0.000006^1^ (Fig. 2).

In detail, Def-RTs were significantly longer if the definition was phrased in abstract (Ad, M = 2038, SD = 518) than concrete terms (Cd, *M* = 1956, SD = 529) and if the item involved was a concrete (Cw, *M* = 2030, SD = 530) vs. an abstract word (Aw, *M* = 1964, SD = 518) (e.g., “flag” rather than “philosophy”). The latter, somewhat counterintuitive, finding is better understood if the Word x Definition interaction is considered. Post-hoc tests showed that when concrete concepts were presented, participants spent more time reading their definitions if these were phrased in abstract (*M* = 2094, SD = 517) than concrete terms (*M* = 1965, SD = 538,* p* = 0.0001). This difference in Def-RTs was not found for abstract concepts (Ad, *M* = 1981, SD = 516; Cd, *M* = 1946, SD = 522 *p* = 0.13). Inspection of the three-way interaction (Fig. 2) further specified that when concrete concepts were presented, longer Def-RTs to abstract than concrete definitions emerged in all participants (High, *p* = 0.0001; Equal, *p* = 0. 003; Low, *p* = 0.04). Conversely, when abstract concepts were involved, a significant difference in Def-RTs was found only for participants in the Low condition (*p* = 0.0002).

### Response time (RT)

On average, participants required approx. 1 s (*M* = 1019, SD = 340) to decide whether the word and the definition matched or not. The ANOVA with Word (Aw, Cw), Definition (Ad, Cd), and Pair (Matching Pair MP, Not Matching Pair NMP) as within-subject factors, and Opponent (Opponent + , Opponent−) and Proportion (High, Equal, Low) as between-subject factors showed main effects of Word, F(1,76) = 75.24, *p* = 0.000001, Definition, F(1,76) = 23.98, *p* = 0.00001 and Pair, F(1,76) = 4.82, *p* = 0.03. The interaction, Opponent x Proportion x Word x Pair was also significant, F(2,76) = 6.00, *p* = 0.004^2^.

**Fig. 3:**
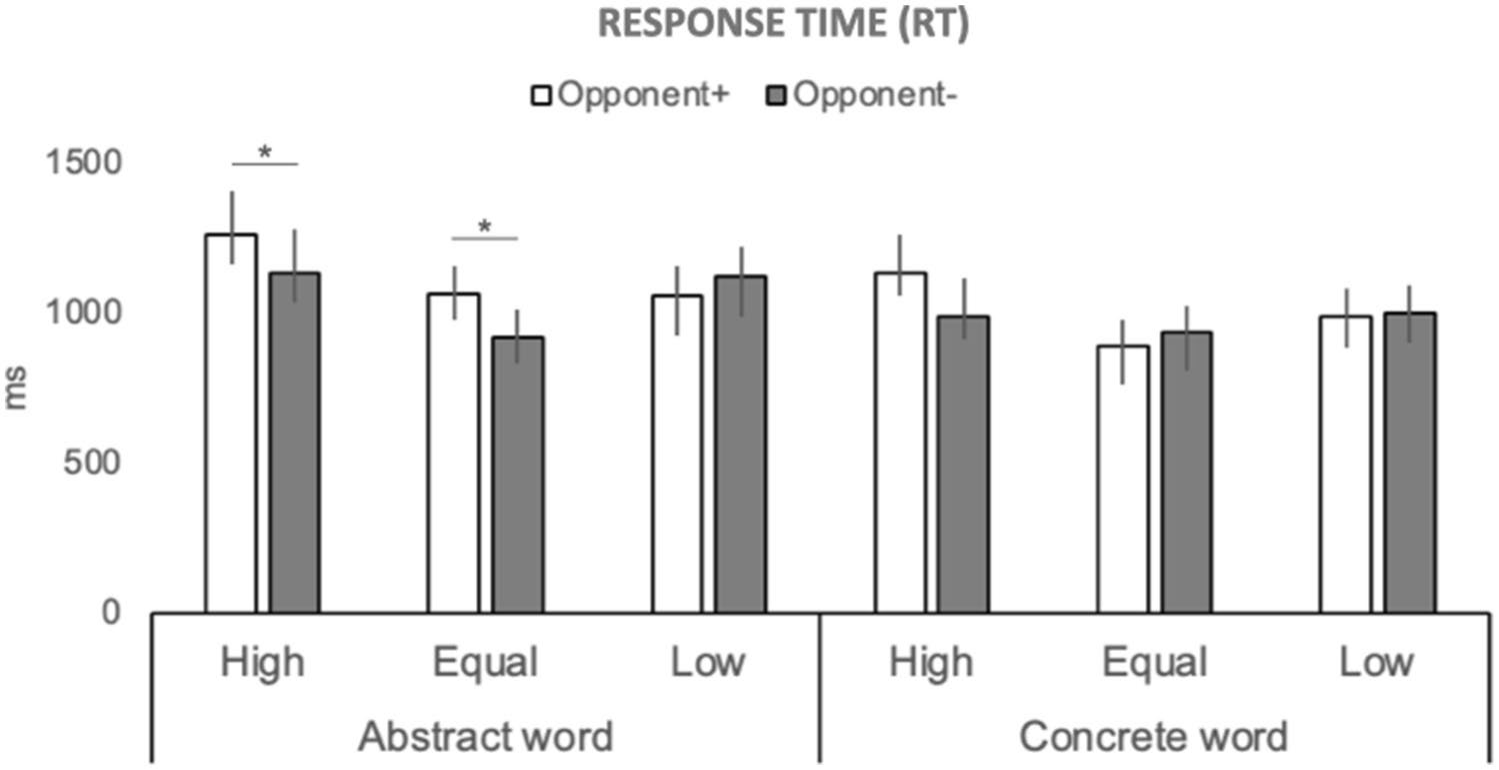
Time to decide whether definition and word match or not (RT). RT was the time between the appearance of the word and the second keypress, i.e., the keypress aimed at responding to whether the definition and word did match or not. Data for Matching trials are reported (please refer to Table 3 for data on Not Matching trials). Significant differences emerged between the responses of participants assigned to the Opponent + (white bars) and the Opponent− (grey bars) conditions, but only if the proportion of ‘response’ trials was relatively high (High and Equal conditions) and if decisions referred to abstract words. No significant differences emerged in the case of concrete words (RTs not presented). Labels as in Fig. 2. Whiskers refer to standard errors; asterisks indicate significant comparisons (*p* < 0.05)

In detail, RTs were significantly longer if the presented words were abstract (Aw, *M* = 1083, SD = 420) compared to concrete (Cw, *M* = 965, SD = 359), and if the definition was phrased in the abstract (Ad, *M* = 1070, SD = 406) rather than concrete terms (Cd, *M* = 978, SD = 378). Besides, RTs were significantly longer for matching pairs (MP, *M* = 1044, SD = 410) compared to not matching pairs (NMP, *M* = 1004, SD = 378). Post-hoc tests relative to the higher order interaction (Opponent × Proportion × Word × Pair) provide some insight into how manipulating the competitive component and fairness of social support affected participants’ responses. Firstly, as reported in Table 3, RTs for Aw were significantly longer than those for Cw in most conditions, notable exceptions being participants in the 2-players setting assigned to the Low condition. Differently from the remaining participants, these volunteers responded with comparable speed to both abstract and concrete words on both MPs and NMPs trials (Table 3). Secondly, participants assigned to the 2-players setting showed significantly longer RTs than participants performing in the 1-player setting–but only when responding to abstract concepts and receiving a favorable or fair number of response trials (Fig. 3). Namely, a difference between ‘solo’ players (Opponent−) and participants assigned to the more competitive environment (Opponent +) emerged only for individuals performing in the High (MP, *p* = 0.04; NMP, *p* = 0.0002) and Equal (MP, *p* = 0.001) condition. Note that this difference was found only when dealing with abstract words. No such effect emerged when concrete concepts were presented (all *p* > 0.05) nor was found in participants assigned to the Low condition (Fig. 3). In addition, when responding to abstract concepts, participants in the 2-players, High condition were significantly slowed down compared to participants performing against an opponent but assigned to the Equal (MP, *p* = 0.0004; NMP, *p* = 0.0002) or Low condition (MP, *p* = 0.0002; NMP, *p* = 0.0003).

**Table 3:** Average RTs (and SD) for participants in the six experimental conditions

	Matching pairs (MP)		*p*	Not matching pairs (NMP)		*p*
	Aw	Cw		Aw	Cw	
Opponent +						
High	1262 (510)	1135 (485)	0.01	1247 (496)	1019 (251)	0.0002
Equal	1064 (361)	890 (268)	0.002	926 (295)	816 (183)	0.02
Low	1057 (380)	988 (354)	0.41	1085 (419)	1018 (432)	0.29
Opponent−						
High	1137 (287)	989 (227)	0.004	1029 (319)	926 (247)	0.007
Equal	918 (283)	937 (434)	0.84	988 (350)	869 (336)	0.001
Low	1121 (417)	1002 (346)	0.05	1118 (381)	966 (295)	0.01

RT was the time elapsed between the appearance of the word on the screen and the second key press. It is assumed to reflect the time needed to reach a decision on whether the word matches the presented definition. Mean values for participants assigned to the different conditions are separately reported for pairs involving abstract (Aw) and concrete (Cw) words and requiring either a ‘match’ (MP) or a ‘not match’ response (NMP). The reported p-values refer to the comparisons between abstract and concrete words.

### Debriefing session

At the end of the experiment, all participants answered a short questionnaire aimed at assessing their reactions. Overall, participants found the task quite simple (67% of raters selected the option ‘easy’; 14% the option ‘very easy’), and not at all annoying (in relation to the statement ‘by the end of the task I felt very annoyed’, 67% of raters selected the option ‘totally disagree’ and 17% ‘disagree’). In fact, only a minority (4%) considered the possibility of quitting before the end of the session. When asked to rate their performance, most volunteers agreed with the statement “I think my performance was good” (57% of raters were ‘in agreement’, 20% were ‘totally in agreement’, or noncommittal, 16%). Participants in the Opponent + groups were further asked to describe their performance in relation to the other player, i.e., they were asked whether they agreed with the statement “I imagine the other player did better than me”. It emerged that 80% of participants in the Equal condition selected the noncommittal reply (‘neither agree nor disagree’), whereas this option was chosen by less than half of the participants in the High and Low conditions. In fact, 60% of participants in the two ‘unbalanced’ conditions expected the other participant to have been either more or less successful than themselves.

### Summing up

Participants were generally very accurate in their responses (~ 95%). All spent approximately 2 s reading the definition (Def-RT) and 1 s deciding on their response (RT), confirming that they complied with the instruction to call for the word only once they had read the definition. Both variables were affected by the abstractness of the stimuli, the presence of a competitor and/or the proportion of ‘response’ trials assigned to the participant (High, Equal, Low). Time spent on the definition (Def-RT) was longer if the definition was phrased in abstract compared to concrete terms. In the case of concrete concepts, this was true for all conditions, whereas in the case of abstract words this difference was found only for participants in the Low condition. RTs to abstract words were significantly longer than those to concrete words in all cases except for participants assigned to the Low condition, particularly those in the 2-players setting, i.e., participants that were apparently neglected or forgotten by the experimenter. Besides, for abstract words only, RTs of participants in the 2-players setting were significantly longer than those of participants playing alone—but only if assigned to the High or Equal condition, i.e., if they were seemingly supported or treated fairly by the experimenter. Finally, when asked about their subjective reactions, the volunteers assigned to the two unbalanced conditions openly reported to have expectations of their opponent’s performance, suggesting that they clearly responded to the interpersonal nature of the task.

## Discussion

Results of the current experiment indicate that abstract stimuli present a selective sensitivity to the manipulations of social context applied to the decision task. Both the level of competitive involvement and degree of social support were varied here, the former by assigning participants to conditions that included vs. not included a direct competitor, the latter by varying the number of response-rounds the experimenter assigned to the participant. Together, these manipulations created situations in which participants were expected to perceive more (or less) support from the experimenter.

Responses to the debriefing questions confirmed that participants in the 2-players setting that had been assigned to the ‘unbalanced’ conditions (High, Low) interpreted the task as social interaction, as expected by the type of manipulation applied. In fact, more than half of them offered an estimate of the other player’s performance vs. their own, which was not the case for participants in the Equal condition, who did not volunteer any opinion on the performance of their opponent. This differential behavior indicates that when a bias was introduced in the competitive context, some form of relational awareness was promoted in the participants, as shown by their appraisal of the other player’s behavior. Thus, implicitly as it may be, the favor/disfavor of the experimenter selectively impacted the performance of these participants but not of volunteers in the Equal condition, who remained noncommittal on the other player’s performance (as could be expected by a fair competition with no overt feedback on what the other player is doing). Given that the question required a comparison with the other player (speculating on whether “the other did better than me”), i.e., an operation relying on a change in perspective that taps into social cognition, the competitive and interpersonal/social nature of the task was evidently at work here.

Intriguingly, manipulation of the social context was especially effective when abstract items were involved. As can be seen in Figs. 2 and 3, both time to read the definition (Def-RT) and the time to decide on its match with the target-word (RT) were significantly longer whenever an abstract—but not a concrete—element (definition or word) was involved. Differences in processing concrete vs. abstract concepts have been previously reported in the literature. In this respect, our data reproduce the well-known concreteness effect, namely the advantage of responding to concrete vs. abstract items, which is typically observed in language and memory tasks (e.g., Paivio, 1991; Schwanenflugel et al., 1988; Taylor et al., 2019). The novel finding is that such effect was not limited to words but extended to sentences, as indicated by the effects reported for Def-RTs (see also Borghi & Zarcone, 2016, for a similar result).

### Effects of describing concrete concepts in abstract terms

In the case of Def-RTs, longer latencies were recorded when the definition described a concrete item using a theoretical framework or taxonomical relations (abstract definition) compared to when it used real-world examples or perceptual attributes (concrete definitions). This finding emerged in all participants, regardless of the condition they were assigned to and could reflect a general difficulty in retrieving concrete concepts when presented within a more abstract framework. To our knowledge, this is the first time this phenomenon is reported. We hypothesize that the observed lengthening of Def-RTs could depend on the fact that when definitions are phrased using taxonomical relations or theoretical descriptions, they are less likely to elicit the physical and perceptual features that are most relevant to concrete concepts. Thus, retrieval of the corresponding concept may become a more indirect—and lengthy, process. Interestingly, only participants assigned to the Low condition spent a longer time on the definition also when abstract concepts were involved, i.e., when the format in which the definition was phrased was compatible with the type of concept it referred to. A possible reason for this unique behavior could be that these participants were assigned to a globally “unfavorable” condition. Indeed, compared to what would be expected by a fair distribution of “response” trials (Equal condition), these participants received less opportunities to gain points. Consequently, when the concept was phrased in a more complex format, their motivation might have been affected, possibly inducing them to be more careful in their assessment of the definition (to minimize the risk of errors).

### Effects of varying social context on decision time

When RTs are considered, several effects emerged. A general increase in latencies was found for responses to Matching than Not Matching Pairs. This delay could be ascribed to the fact that in Matching Pairs participants engaged in a confirmatory process prior to responding (e.g., a comparison with some prototypical definition they hold in memory)—a process that could be waived in the case of Not Matching Pairs. Importantly, data on RTs further informed on the peculiar interaction between the abstractness of the stimuli and manipulations of the social context applied. Influence of the competitive setting was particularly evident when participants were assigned to a patently favorable condition, namely when they were called to respond in a high proportion of the trials (condition High) or when there was a fair balance between “response” and “wait” trials (condition Equal). In these cases, the supposed presence of an opponent made a significant difference by further lengthening RTs as opposed to when participants were assigned the same amount of “response” and “wait” trials but were told they were playing alone. Remarkably, this difference is only related to the processing of abstract material, supporting the idea that social context influences abstract concept processing. This finding may seem at odds with the idea that the acquisition of abstract concepts relies on the contribution of “helpful others”. However, according to WAT, others can not only be informative and supportive but may also contribute to negotiating the word's meaning, reflecting different positions and points of view (Borghi, 2022). Notably, the meaning of abstract concepts is less univocal and much more dynamic than that of concrete concepts. Accordingly, other individuals may help in understanding novel information but may also contribute contrasting definitions from our own—as may arise from differences in experiences, age, or culture. In either case, the processing of abstract concepts will be nevertheless influenced by the social nature of the interaction. This latter aspect could have been even more relevant here considering that adults were tested i.e., individuals whose verbal abilities are assumed to be fully developed, and who would be more frequently exposed to experiences whereby concepts’ meanings are negotiated rather than explained. Indeed, the social interaction explored here was not aimed at explaining concepts’ meaning but at creating a social environment that could be more (or less) favorable to the responder (namely, the experimenter assigned response turns, but he/she did not provide cues to the solution of the verbal task). Yet, it is remarkable that manipulations affecting social context without a genuine interaction still showed a significant and selective influence on abstract concepts. In fact, further aspects of social cognition (such as empathic concern) may be relevant to the processing of abstract material. In this respect, we offer two main reasons for the lengthening of RTs that was observed in participants in competitive, socially favorable conditions: one pertaining to the complexity of the material, and one to the co-occurrence of various social factors.

### Role of complexity of abstract concepts

The simplest explanation could reside in the higher degree of complexity required to cognitively process abstract concepts. Although the concept of abstractness itself still lacks an unambiguous and unitary definition (see for instance Desai et al., 2018; Vargas & Just, 2020; Conca et al., 2021), it remains that compared to concrete concepts, abstract items do present with a more complex pattern of representations in the brain. According to a recent proposal (Buccino et al., 2019), this different degree of functional complexity would depend on the fact that only abstract concepts possess the peculiar characteristics of being: (i) effector-unspecific, i.e., related to different contextual representations (cf. ‘freedom’, as in ‘f. to leave/f. to speak/f. to think’, etc.); (ii) multi-systemic, since they simultaneously recruit more than one brain system (other than the sensory-motor one), and (iii) dynamic, because their meaning is susceptible to vary with both time and experience. For example, the concept of ‘freedom’ varies considerably in the framing perspective of an adolescent as opposed to that of an elderly individual (Buccino et al., 2019). The Opponent + High condition was not only socially favorable to the responder but also represented the most effortful situation because participants were called to respond in most of the trials. Thus, it could be that when abstract material was presented, its intrinsic complexity—evoked by the basic, verbal (i.e., phonological/lexical) processing of the stimuli—could have delayed the responses, particularly when participants were exposed to a fast pace of response. In other words, the higher latencies observed in this group for abstract material could be accounted for by the more taxing cognitive processing involved, a phenomenon that could have been enhanced in the more demanding experimental conditions.

Although plausible, an account exclusively in terms of higher cognitive effort when dealing with abstract concepts does not entirely explain our findings. If we assume that the intrinsic complexity of abstract material adds to the cognitive effort required by the task, we should expect some linear trend in RTs distribution across abstract conditions as a function of the amount of ‘response’ trials assigned to the participants. Namely, the more frequent the response turns, the more demanding the task would become, and consequently the longer should be the latency of response. This was only partly found: although participants in the High groups were generally slower, participants in the Opponent-, Equal group (i.e., those responding in 50% of trials) were comparatively faster than those responding in the 33% of trials (Low groups), i.e., those performing at an overall less demanding pace. These findings, if considered in light of the intrinsic competitive nature of the task, rather suggest that motivational factors probably contributed to the reported interactions, eventually adding to the effects of the complexity of the material. We propose that at least two such factors could be involved.

### Role of social factors: chocking under pressure

As specified by the instructions given to participants, response turns were assigned by the experimenter—entirely at his/her own discretion. Consequently, it can be hypothesized that a condition that clearly favored participants may have seen them more prone to pondering answers and investing in doing their very best to avoid that the advantage granted appeared as ‘undeserved’. Paying too much attention to performance may thus have delayed responses, due to what has been described as ‘choking under pressure’, namely a specific decrement in performance induced by the stress of the situational pressure (Baumeister, 1984; Baumeister & Showers, 1986). Within this framework, performing more poorly than expected may result from the fact of being under observation (monitoring pressure) as well as from the desire to achieve success (outcome pressure). Therefore, individuals may become more prone to distraction (since attention would be diverted to something not directly related to the task, e.g., the consequences of a failure) or–conversely–more prone to excessive self-focusing. Both attitudes would obviously interfere with performance (DeCaro et al., 2011; Lewis & Linder, 1997; Liao & Masters, 2002). The decrement in performance reported here whenever an opponent was implied would be in line with the hypothesis of an increased burden created by the more competitive pressure of the task compared to conditions in which participants were informed that they played alone.

### Role of social factors: perceived inequality

Within the frame of intervening motivational factors, a second possible factor could have arisen from the asymmetrical distributions of response turns applied here. Albeit studied mostly in the context of economic games and in relation to payoff, perceived inequality appears to be a powerful stressor than can exert significant effects on behavior (Fehr & Schmidt, 1999; Loewenstein et al., 1989; Shapiro et al., 2017; Yu et al., 2014). In our case, the advantageous condition of being asked to respond in more than half of trials could have been affected by the negative feeling of competing against an unfairly treated opponent as well as that of being the recipient of a discriminatory benefit. The potential stressing effect of competing against another player in a context of strong discrimination may thus account for the longer RTs always observed in the Opponent + High group. The reason why this supposed feeling of inequality failed in producing detectable effects also in the specular situation of unfairness (Opponent + Low) could be due to the rather sparse involvement of the participants in this condition, since in this case only about 30% of the responses were assigned to the participant. Accordingly, it is possible that the relatively reduced cognitive effort of working at a slower pace and in a less demanding setting may have compensated for the adding cost of the perceived inequality.

Presently, we cannot tell which of these factors are more relevant—this could be better addressed by further research—nor can we exclude that the current results arise from a combination of the suggested cognitive and motivational factors. It is altogether possible for example, that when dealing with abstract material participants could have been implicitly induced to adopt a ‘softer approach’ in making their judgments to better comply with the more pressing demands of the task or that they may have been affected by a combination of factors. As a matter of fact, it is plausible that the fact of working with the more engaging items in a higher density of trials may have caused one or more of the abovementioned motivational responses to interact, thus producing the selective lengthening of response observed in these conditions.

### The overarching role of context

Be as it may, it remains that only abstract concepts showed a specific sensitivity to the experimental manipulation applied here. An alternative, not mutually exclusive, explanation could be proposed that suits both the peculiar nature of abstract material and the social nature of the task used. Presently, a univocal definition of what could be labeled as ‘abstract’ is still lacking, which undoubtedly highlights the elusive and multifaceted nature of this material. In fact, abstract concepts can tap into more than a single dimension, engaging more than one system whenever their processing occurs (e.g., Desai et al., 2018), and can be characterized by a different degree of conceptual features including verbal, social, introspective, and affective information (see for instance Harpaintner et al., 2018). As pointed out in a recent systematic analysis, abstract concepts are thus part of a multidimensional category that in the brain relates to distinct neural networks depending on the type of knowledge they refer to (Conca et al., 2021). In line with Buccino et al. (2019), we maintain that the complexity of abstract concepts could be reduced by contextualization. Context would provide the information required to resolve the complexity of the ‘experiential cluster’ to which abstract concepts associate, allowing the retrieval of the precise subset of experiences that more appropriately defines them according to the current situation (Buccino et al., 2019). For example, if we consider once more the concept of ‘freedom’, it becomes clear that disentangling between different instances as in ‘f. to leave/f. to speak/f. to think’, etc. is almost impossible without a clear contextual definition. The pattern of results observed here for abstract concepts could similarly reflect their strong ‘contextual dependency’. Namely, the processing of abstract material may be specifically affected if the functional systems required for their specification are also responding to other contextual demands, such as those elicited by social interactions. Since abstract concepts lack the support of an objective physical counterpart, social skills may be needed to correctly grasp or express the intended meaning because the exact significance of a concept may change based on gender, age, status, etc. Gaining insight about the speaker (or the listener) may thus be needed to correctly appreciate the concept’s meaning. In neural terms, social cognition depends on the activity of a specific network responsible for the ability to represent and interpret others’ behavior (Conca et al., 2021; Desai et al., 2018; Vargas & Just, 2020). In view of this social imprinting of abstract knowledge and the need to contextualize abstract material, the current findings can thus be interpreted in an entirely different framework. The selective slowing of responses to abstract items observed in the Opponent + High group (compared to the Opponent- High group) could be viewed as the result of a shared cognitive engagement—a sort of cognitive trade-off—due to the recruitment of the same resources for the purpose of dealing with the fictional competition on one side, and with the need to contextualize the conceptual material presented to solve the task on the other. The increase in response latencies to abstract concepts—specifically in the presence of an opponent—may be because when the interactive situational context included two participants, the cognitive representations claimed to face the game must be partaken with those involved, trial-by-trial, in the judgment task. This interpretation would fit with the idea of a social foundation of abstract concepts, whereby sociality would be the crucial determinant for their acquisition as well as for the situational contextualization of the experiences they relate via language (words as social tools, Borghi et al., 2019). Even if fictitious, the social context determined by the alleged presence of another player selectively affected participants’ performance, replicating in human behavior the peculiar ‘social sensitivity’ of the brain reported by animal studies (see Demolliens et al., 2017).

One last remark could be made with respect to the role played by context in the representation of knowledge. Context can act as a powerful organizing tool. In memory for example, when the context is made available, even if only indirectly, as a scene offering a scaffold for learning material, recall is significantly enhanced (Robin & Olsen, 2019). In this sense, the reconstructive process of remembering (see for instance Binte Mohd Ikhsan et al., 2020) can be considered as a mean to maintain contextual support for stored information. The need for contextualization to sustain a coherent and stable representation of reality could thus be viewed as a general coding mechanism potentially capable of organizing the whole knowledge (Behrens et al., 2018). As such, it is not surprising that a structure such as the hippocampus is seen as responsible for creating conceptual knowledge on an episodic basis (Mack et al., 2018). When viewed as an organizing principle, context could acquire the role suggested by Desai and coll. who wrote that: “for some abstract concepts, exemplars and prototypes are events, and family resemblance is computed over event structures” (2018, p. 12). Being rich in event-based information, social interactions may represent a fruitful mechanism for grounding concepts, as suggested by the similarities observed in brain activity when dealing with sets of abstract concepts and social interaction memories that share the same components (Desai et al., 2018).

### Limitations

It should be stressed that in the present experiment, we did not weigh the relative contribution of the various components that support abstract knowledge (e.g., emotionality) and that could have differently affected the target-word used. As previously mentioned, these components are functionally dependent on the activity of specific and distinct brain networks. Thus, in considering the possibility of a shared cognitive engagement between the interactive context elicited by the task, and the processing of abstract items we cannot exclude that other dimensions, mostly emotional and interoceptive ones, may have also contributed, given their relevance to the experience of a competitive interaction. Consequently, the contextual responsiveness of abstract items could then be thought of as a corollary to their whole complex, multi-composed essence. Albeit reasonable, we believe that the emotional character of words is unlikely to have influenced the present results, for at least two reasons: first, we have deliberately excluded strictly emotional words from the selected ones, and second, recent evidence suggests that abstract concepts are in fact not more emotional than concrete ones (with the obvious exception of abstract emotional concepts; Winter, in press). In a similar way, for the purpose of the current study we used a rather broad definition of social context—that extended to both the interpersonal relation with the experimenter and the presence/absence of another player. A more detailed exploration of how the manifold forms of interpersonal relationships interact with the processing of abstract material goes beyond the scope of the present paper but we hope novel studies will be run that focus on selected aspects of social interactions, as they should provide valuable information.

## Conclusions

To conclude, the data we presented here clearly support the idea that social context is a factor affecting the processing of abstract knowledge. Indeed, as it has been recently pointed out: “during immediate cognitive and affective processing, contextual factors not only have continual influence, but their effects are often substantial” (Barsalou, 2019, p.220). Accordingly, the necessity emerges that experimental paradigms akin to the one we adopted here should be applied to the study of concepts’ nature within the contexts and interactions in which they ecologically are grounded (see Barsalou, 2018, 2020).

## Data Availability

The data that support the findings of this study may be made available from the corresponding author upon reasonable request and under a confidentiality agreement.

## Appendix 1

**Table 4:** Ratings for the set of definitions used in the current study

	Word production	Word/definition association	Concreteness/abstractness
Abstract definitions			
Abstract words	0.62 ± 0.2	5.37 ± 1.7	4.68 ± 1.8
Concrete words	0.73 ± 0.2	5.41 ± 1.9	3.51 ± 2.0
All items	0.68 ± 0.2	5.39 ± 1.8	4.15 ± 2.0
Concrete definitions			
Abstract words	0.65 ± 0.3	5.40 ± 1.6	4.50 ± 2.0
Concrete words	0.86 ± 0.2	5.93 ± 1.5	3.12 ± 2.2
All items	0.76 ± 0.2	5.67 ± 1.6	3.78 ± 2.2

The first column (Word production) shows the proportion of participants (± SD) reporting the target after reading the definition. The second column (Word/definition association) reports mean ratings (± SD) relative to the perceived degree of association between definition and target concept (on a 7-point scale, 1 = no association, 7 = very strong association). The third column (Concreteness/Abstractness) shows mean ratings (± SD) for the concrete/abstract dimension (on a 7-point scale, 1 = very concrete, 7 = very abstract). Values correspond to averages of the ratings obtained for the items included in each category (e.g., 20 abstract definitions of abstract words; 20 abstract definitions of concrete words; 40 abstract definitions in total)

A pre-test was run to ensure that the selected definitions correctly captured the intended content, and that Cd and Ad were perceived as different by the readers. For this purpose, the 80 definitions were submitted for evaluation to 80 naïve university students. Each participant received three forms. One form contained ten definitions and participants were asked to produce the concept/concepts evoked by each of them (Word production). The second form contained ten word/definitions pairs and participants rated on a 7-point scale how well each definition described the associated word (Word/definition association). The third form contained ten definitions and participants rated each of them along the concrete/abstract dimension; a 7-point scale was used, 1 meaning very concrete, 7 very abstract (Concreteness/abstractness). To avoid confounds due to repetitive exposure, different words and definitions were used in each of the three forms. A definition was considered acceptable if it produced the target word in at least 50% of participants and received an association rating above 4 (4 being the mid-scale value). Reasonable synonyms, colloquial terms, morphological/orthographical variants were accepted if they clearly referred to the intended concept. Sixteen definitions did not satisfy these criteria and were modified. A new group of 30 naïve participants (age, *M* = 23, SD = 3, 17 females) evaluated the sixteen modified items by means of the same protocol used for the first group. After this second test, all definitions evoked the target word in at least 60% of raters and/or were rated above the mid-scale value. As for the concrete/abstract dimension, average ratings for concrete definitions were significantly lower (i.e., closer to the concrete end of the scale) than those for abstract definitions (*t* = 2.043, df = 39, *p* = 0.058, see Table 4).

## Footnotes

1 An additional 3-way ANOVA was performed with Definition (Ad, Cd) and Group (Opponent + /High, Opponent + /Equal, Opponent + /Low, Opponent-/High, Opponent-/Equal, Opponent-/Low) as within-item factors and Word (Aw, Cw) as between items factor, which basically replicated the reported findings, and specifically the three-way interaction Group, Word and Definition, F(5,90) = 4.60, p = .0001. For the sake of simplicity, the two factors Opponent and Proportion were here treated as one.

2 An additional 3-way ANOVA was performed with Definition (Ad, Cd) and Group (Opponent + /High, Opponent + /Equal, Opponent + /Low, Opponent-/High, Opponent-/Equal, Opponent-/Low) as within-item factors and Word (Aw, Cw) as between items factor. Overall, this analysis replicated the reported findings, although here the interaction Word x Group just failed to reach significance, F(5,90) = 1.93, p = .09. Note that this additional analysis was performed solely on RTs relative to matching pairs (i.e. “b key-press” responses) because in non-matching pairs two different concepts were involved, which could create a confound for this type of analysis. By halving the number of entries however, power could have been reduced.
